# A cyclic learning approach for improving pre-stack seismic processing

**DOI:** 10.1038/s41598-021-87794-8

**Published:** 2021-04-21

**Authors:** Dario Augusto Borges Oliveira, Daniela Szwarcman, Rodrigo da Silva Ferreira, Semen Zaytsev, Daniil Semin

**Affiliations:** 1 IBM Research, São Paulo, 04007-005 Brazil; 2 Gazprom Neft, Saint Petersburg, Russia 190000

**Keywords:** Geology, Geophysics, Computational science

## Abstract

Current seismic processing workflows in the oil and gas industry involve several interactions between different experts to optimize the overall data quality in various tasks, such as noise attenuation, velocity analysis and horizon picking. While many machine learning-based approaches have been proposed to support each of those steps, most of them disregard expert interactions to guide the overall optimization. This paper presents geocycles, a cyclic learning approach that mimics this iterative process, which can be applied to different pre-stack seismic processing tasks. Our method refactor these processes considering training, testing, and evaluation sub-tasks, which allow the selection of samples for greedy sequential processes targeting an overall optimum quality for very large seismic datasets. We present encouraging results showing that a cyclic structure and efficient quality metrics improved overall outcomes in up to 128% for two different seismic processing tasks in comparison to a 1-cycle machine learning approach.

## Introduction

According to Yilmaz^1^, one of the main applications of the seismic method is exploration seismology, i.e., the exploration and development of oil and gas fields. The first stage of the seismic exploration process is data acquisition, which usually requires a source of mechanical waves such as dynamite for land surveys or air guns for marine surveys. The mechanical waves travel into the Earth, crossing strata with different impedances, and then are reflected back to the surface where they are recorded by receivers. These receivers are called geophones for on-shore surveys and hydrophones for off-shore surveys.

Surface, environmental, and demographic conditions may have a significant impact on the quality of the data collected in the field. Typically, the acquisition is carried out under sub-optimal conditions, and a substantial part of the processing workflow is dedicated to attenuate the noise and enhance the signal. The main steps of seismic processing are *preprocessing*, *deconvolution*, *stacking* and *migration*, although this order may differ depending on the case. Preprocessing involves amplitude correction and noise attenuation. Then, deconvolution is performed along the time axis to increase temporal resolution by compressing the basic seismic wavelet. The stacking procedure compresses the offset dimension, which reduces the seismic data volume and increases the signal-to-noise ratio. Finally, migration is performed on the stacked data to remove diffractions and move dipping events to their correct positions^1^.

In recent years, there has been an increasing interest in applying machine learning (ML) techniques to aid seismic interpretation and processing. We have seen many applications of ML in seismic facies analysis^2,3^, horizon picking^4,5^, seismic inversion^6,7^, migration^8,9^, porosity estimation^10,11^, petrofacies classification^12,13^, data interpolation^14,15^, denoising^16,17^ and stacking^18,19^. This shows that the relevance of machine learning in the oil and gas industry is growing in a similar way to other industries. Most of these works have the potential to increase the accuracy and reduce the time necessary to perform typical tasks in seismic processing. However, there is still an almost unexplored space in the literature on how such methods can be deployed in practice. The work of Tschannen^20^ represents a first step in that direction by analyzing deep, transfer, supervised and unsupervised learning in the context of seismic processing and interpretation. Nevertheless, the work still lacks a unified framework to guide the optimization process.

To the best of our knowledge, this is the first work to propose a robust methodology—named *Geocycles*—to assess and improve the performance of machine learning methods for seismic processing and guide their optimization in a cyclic approach. Our approach creates an ensemble of models that jointly and efficiently tackle very heterogeneous datasets by specializing models to parts of the data selected using solid quality control metrics iteratively. Although it has similarities to ensemble learning^21,22^, our approach is different in essence. While ensemble learning typically uses voting to combine the predictions of each model, here we use the concept of “useful overfitting”, in which each model becomes a specialist in a specific region. Similarly, if our methodology is used with a human in the loop, it could resemble an active learning approach^23,24^. However, unlike active learning, we are not interested in gathering new labeled data to improve a single model but rather to build specialized models for regions with different characteristics. Among other benefits, Geocycles can be easily coupled with current workflows, providing a framework to drive the training of machine learning models based on clear quality metrics that not only may produce performance gains in comparison to traditional machine learning approaches but also fits nicely to recent human-in-the-loop and ML explainability requirements.

Although the method can be theoretically applied to any machine learning algorithm in pre-stack seismic processing, in this work, we present two use cases that demonstrate its applicability to ML used in different steps of seismic processing. In the first one, we discuss the preprocessing step, focusing on ground roll noise suppression. In the second use case, we apply our method to the velocity analysis step, which is crucial for stacking.

## Method: geocycles

In the oil and gas industry, the seismic processing workflow typically comprises several interactions between different experts to perform specific tasks targeting consistency and data quality assurance at each step. Such a characteristic enables the modeling of a machine learning-based seismic processing from an iterative optimization perspective. We build on that to propose a cyclic learning framework called *Geocycles* for optimizing seismic processing tasks. The idea is to mimic the expert interaction process in which the user reprocesses samples with poor results and sets aside the ones that already have sufficient quality, aiming at an overall optimal outcome.

**Figure 1:**
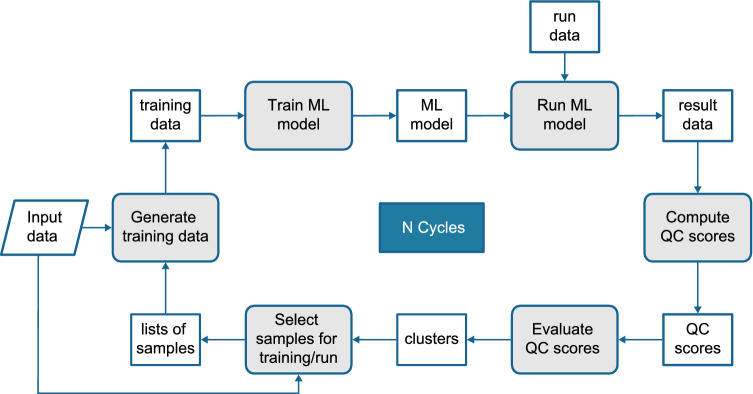
Geocycles is composed of machine learning tasks, score computation and evaluation, and sample selection.

Figure 1 presents the Geocycles framework which is composed of machine learning tasks, quality score computation and evaluation, and sample selection. The details of each step will depend on the specific seismic processing task that Geocycles will optimize. The overall workflow can be summarized as follows. First, the method generates the data to train the ML model to perform the specified task, based on an initial sample selection rule. After the training is complete, the model is ready to process the data. Then, quality scores are assigned to these results. Finally, according to the computed scores, a selection process defines which samples should go to the next cycle and which should not. Notice that our greedy strategy evaluates the outcome quality iteratively and trains new models for data that presented low-quality results. In the following, we describe the steps of the workflow in more detail.

### Selecting initial samples

With a task at hand, the first step for many machine learning solutions is typically to select the data to train the model. The criteria for this selection vary significantly and also depend on specific characteristics of the ML model, such as the number of annotated samples required for training. A common approach is to select a few examples randomly, considering all the available ones. In our framework, the sample selection step is also responsible for defining the data that the model will process in a given cycle (referred to as *run data* in Fig. 1). We highlight that sample selection is required at every iteration of the workflow. However, the initial criterion might be different from the one used in all subsequent cycles, since at the beginning of the process, the data quality scores are not available yet.

### Machine learning task

Once the data is organized, one needs to specify a machine learning method to solve the seismic processing task of interest (e.g., noise attenuation). This stage depends on the problem itself, with several possible solutions and a wide variety of requirements and human decisions involved in the process. For instance, in the use cases we present a solution using conditional GANs (Generative Adversarial Networks)^25^ for noise attenuation.

Note that the samples selected for training should be preprocessed according to the specified task and ML model. In other words, the step *Generate training data* in Fig. 1 must include the necessary data preprocessing for the particular ML solution defined. When the model is trained, the data to be processed (*run data*) is finally fed to the model, and the results for the given task generated. Data generation, model training and execution usually comprise a typical ML solution pipeline. Here, we propose a few more steps to enable a cyclic optimization scheme.

### Computing metrics and compiling scores

After running the ML model, quality check (QC) scores are computed and associated with the outcomes. The goal of the automatic evaluation of results is to characterize them by important features that will be monitored at the end of each cycle. Such features (or *metrics*) should reflect the desired characteristics in the typical expert evaluation process for the given seismic processing task. For example, if the geoscientist analyzes reflector amplitudes as a quality standard for the data, then they are an adequate feature to monitor at the end of each cycle.

After the computation of the outcome metrics, we evaluate how close they are to a given reference that can be extracted from reference data or defined as a theoretical target. This way, we transform metrics into scores and enable an automatic cyclic optimization scheme. Scores are expected to increase as metrics get closer to the desired values.

### Evaluating the scores

Evaluating the samples in our approach means grouping them by their QC scores to assign a semantic meaning that characterizes groups of samples. Such groups can help define strategies for selecting the data for the next cycle and the stopping criteria. Additionally, they may make it easier for an expert to analyze the results at each cycle.

To do that, we build a scale that defines the criteria for grouping samples into three clusters of quality, namely: *good*, *average*, and *bad*. The cluster *good* is the one with the best average score, *bad* is the cluster with the worst average score, and *average*, the one in between. The scale can be predefined, with fixed ranges of QC values for each group. For example, the group *good* incorporates samples with QC scores above 90%. Another possibility is to fit a clustering algorithm (e.g., K-means) to the QC scores. The latter approach has the advantage of flexibility, as the groups are defined according to the QC scores available.

### Selecting samples for the next cycle

To complete the workflow, we must define how to proceed to the next cycle. More specifically, we need to establish which data samples should be reprocessed in the next iteration and determine the stopping criteria.

As for the sample selection, a possible criterion is to pick samples from the group *bad* for training and reprocess only the data in the groups *bad* and *average*. In this case, we are assuming that samples in the cluster *good* already have sufficient quality and should not be re-evaluated. This criterion ensures that the cycles not only improve the overall quality but save processing time in the following iterations.

Finally, many stopping conditions are possible, such as a maximum number of cycles that can be executed. Another option is to evaluate the evolution of the clusters throughout the process. For instance, one could monitor the number of samples that fall into the group *bad*, and if it is too small, the execution could be interrupted.

## Use cases

We selected two use cases to show the potential of Geocycles for different pre-stack seismic processing tasks: velocity picking and ground roll noise suppression. For each of them, we present the deep learning method implementing the task, the metrics and scores used for triggering the cycles, and the parameters for score evaluation and sample selection.

### Velocity picking

The goal in the Velocity Analysis (VA) use case is to assist the geoscientist in the task of velocity picking for Normal Moveout (NMO) correction. In this process, given an input CDP gather, a velocity function is picked and then used to correct the gather regarding the vertical deviations caused by the increasing offset. In a traditional workflow, the geoscientist manually picks the velocities by analyzing the velocity spectrum of selected gathers, and the picked velocity functions are interpolated for the rest of the pre-stack dataset. This procedure is usually a time-consuming, trial-and-error process^26^.

**Figure 2:**
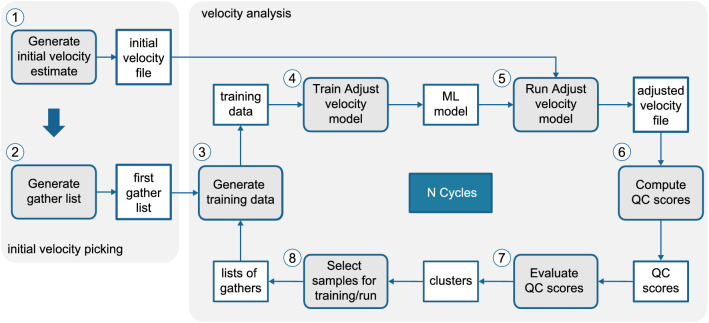
VA use case. The numbers show the order of execution and the gray blocks highlight the sequence of operations that composes the geocycle.

Here, we consider the automatic methodology proposed in Ferreira et al.^26^ It consists of two main parts: (1) *initial velocity picking* and (2) *velocity adjustment*. The first one determines an initial guess for the velocity functions, and the second relies on deep CNNs to adjust the initial estimates. The network receives as input an image containing a subset of a given gather. Then, it predicts the velocity adjustment that should be applied so that the corrected gather presents a flat pattern (no offset deviation). The second part of the pipeline defines a sequence of operations that can be executed in cycles to improve the results. Figure 2 shows the main steps of the workflow, which we detail in the following sections.

#### Initial velocity picking

Different approaches for the initial velocity picking are possible. Ferreira et al.^26^ uses an automatic method based on velocity spectrum maxima picking, followed by a semblance-weighted spline fitting process and a Savitzky–Golay filter to smooth the velocity functions. A supervised alternative, which is used in this work, is to interpolate the given reference velocity functions and use the average of all interpolated functions as the initial picking (Fig. 2, step 1). Since the velocity adjustment part is supervised, we assume that such reference velocity functions will be available.

Next, we select some gathers as initial training data. The specific number is a parameter of the algorithm ($N_{train}$), and in this work, we set it to ten. Step 2 in Fig. 2 is responsible for generating the first training gather list that will be used in the first cycle execution. We randomly select $N_{train}$ gathers, giving preference to those that have a reference velocity function available. Assuming that these velocities are picked by an expert, this approach guarantees that our first training list will have gathers with the best quality velocity functions.

#### Velocity adjustment

The second part of the pipeline comprises steps 3 to 8 in Fig. 2. When step 8 ends, its output feeds step 3, closing the cycle. In summary, first, we generate the training data for our neural network model (step 3), considering the first training gather list. Once the model is trained (step 4), it can be used to adjust the initial velocities (step 5). Finally, we compute the QC scores for the adjusted velocities (step 6) and analyze the results (step 7). The last step is responsible for generating a new training gather list to be fed back to step 3, in the case the stopping conditions are not reached yet.

Since our focus in this work is on the Geocycles methodology, for details about steps 3 to 5, we refer the reader to the method presented by Ferreira et al.^26^. Essentially, those steps generate the training dataset and train a CNN based on the Xception architecture^27^ to predict a velocity adjustment in m/s, given a subset of the seismic gather image.

**Calculate QC**: Once an adjusted velocity function is obtained, step 7 is responsible for calculating the QC scores for the corrected velocities. We considered two possible QC scores to assess the quality of the results—*difference score* and *semblance score*—but only the results with the difference score will be reported in this work. The difference score measures the difference between the adjusted velocity function and the reference. The method computes the average velocity displacement between the reference velocities and the adjusted ones, which results in a distance measure in terms of velocity units (m/s). For this particular score, the lower the values, the better. This is an exception to our previous definition of quality scores since there is no clear upper bound for distance measures.

It is possible to use a weighted average, to give more importance to velocity points corresponding to lower time values (shallow part). Velocity typically increases with depth in a seismic dataset, which makes the errors made in the deep part less relevant than the ones made near the surface. More specifically, the weight values go from 1 to 0, from the lowest to the highest time value. The user can choose to use the regular average or the weighted version. In this work we use the weighted average. Equation (1) presents the difference score definition:

1 $$\begin{aligned} \overline{\Delta V} = \dfrac{\sum _{i=1}^{N}w_i |V_{REFi} - V_{ADJi}|}{\sum _{t=1}^{N} w_i} \end{aligned}$$

where $V_{REFi}$ is the velocity point *i* of the reference velocity, $V_{ADJi}$ is the velocity point *i* of the adjusted velocity, *N* is the number of time-velocity points in both velocity functions and $w_i$ are the weights. Note that the weights can be all set to 1 (standard average) or set to values between 0 and 1, with the highest values multiplying the first velocity points (weighted average).

**Evaluate QC**: In this use case, we employ the K-means algorithm to separate the data into the groups *bad*, *average*, and *good*. As already mentioned, if the QC is given by the *difference score*, the *best score* means the *lowest value*. We highlight that the groups are defined with respect to the QC scores of the first cycle, that is, the values characterizing good, average, or bad results depend on the evaluation of the first outcome. This means that, in our experiments, the fitted K-means is used in the next cycles to separate new scores into the three groups already defined. Nevertheless, one could update the scale on demand.

**Select samples for the next cycle**: In the VA geocycle, we consider that samples that fall into the group *good* already have satisfactory results, and therefore, will no longer be processed in the next iterations. The list of gathers to adjust in the next cycle will contain only the average and bad samples.

For this use case, we randomly select $N_{train}$ gathers from the top 100 in the group *bad* to compose our new training list. Step 8 (Fig. 2) also updates the velocities and QC scores from previous cycles: it replaces the old velocity functions and the old scores if the current scores are better. This way, we guarantee that we always keep track of the best scores and velocities that were obtained during the entire process.

This last step is also responsible for deciding if the cycles should continue or not. In this use case, we selected two stopping conditions. The first one considers the number of gathers that fall into the group *bad*: if it is lower than the required number of gathers for training $N_{train}$, then the cycles should stop. The second condition considers the percentage $P_{good}$ of gathers that go to the group *good*. In this case, if the number of samples going to the group *good* in the current cycle represents a small percentage of the total number of gathers, the cycles should stop. The idea is that little improvement would be achieved if we continue to run the process. $P_{good}$ is a parameter of the method, which we set to 3% for this work.

### Ground roll noise suppression

For the ground roll noise suppression (NS) use case, we used the method proposed by Oliveira et al.^28^ as the main backbone in our cyclic optimization scheme. Therefore, in the remainder of this section we will consider the masks for noise detection as computed in that work and focus on the cycle dedicated to improving noise suppression itself.

**Figure 3:**
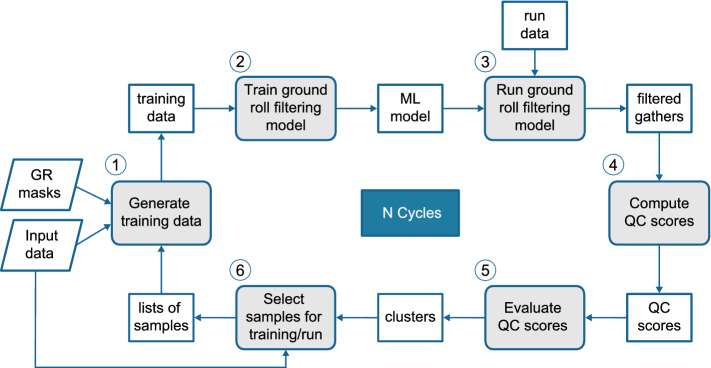
NS use case. The numbers show the order of execution and the gray blocks highlight the sequence of operations that composes the geocycle. Ground roll (GR) detection masks are inputs to this geocycle.

Figure 3 shows the geocycle for filtering ground roll noise. Note that, similar to the VA use case, the geocycle contains all the necessary steps: ML training and execution tasks, QC computation and evaluation, and sample selection.

We selected conditional generative adversarial networks (cGANs) as the ML model to filter ground roll noise, in the same way as described by Oliveira et al.^28^. Their methodology uses paired noisy/noise-free synthesis and disregards supervision or reference data^28^. Hence, the NS geocycle implements a self-supervised method for filtering ground roll noise. The filtering approach enables cGANs to hold the high-frequency information related to the signal, attenuate low frequencies associated with ground roll, and restore fundamental low frequencies related to the signal.

We further consider two types of cycle configurations: one for improving over attenuation, which means avoid suppressing not only noise but also signal, and another for improving under attenuation, which means avoid remaining ground roll noise in the gather. The cycles are identical, but the selection of training samples for the next iterations differ, as detailed in the following.

**Calculate QC**: As explored by Oliveira et al.^28,29^, we selected some of the commonly used criteria in the industry to infer the quality of ground roll suppression, which allows us to inspect the outcome quality disregarding any reference data.

**Figure 4:**
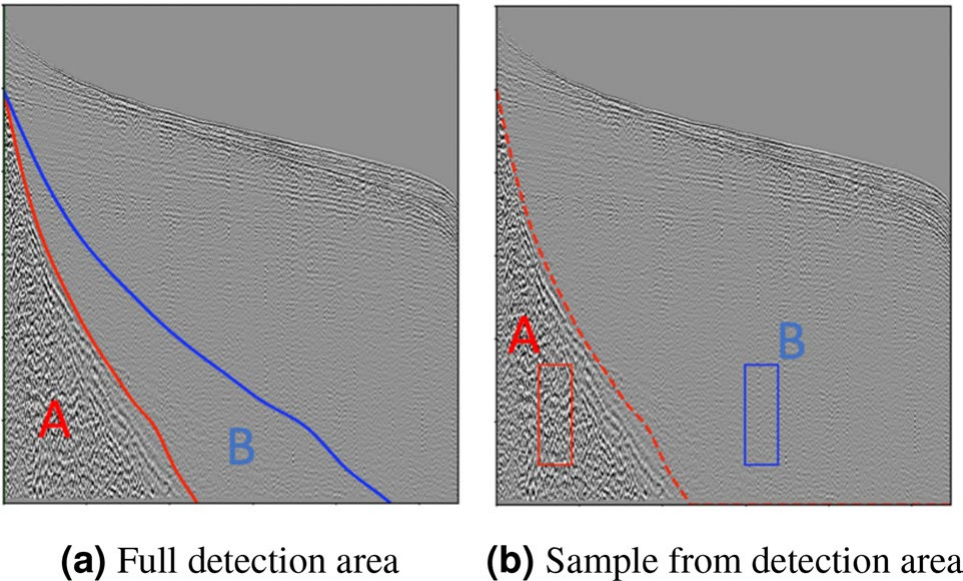
Gather *loci* for computing filtering metrics: region A corresponds to ground roll affected regions and region B corresponds to noise-free regions. (**a**) The detected area and the corresponding mirrored signal area, and (**b**) rectangular samples from ground roll-affected and noise-free areas. Image from Oliveira et al.^28^.

We considered two different *loci* strategies for measuring quality in a contrastive analysis setup, each of them defining a region affected by noise and another region with only valid signal. The first considers the noise region as the full ground roll mask, and the signal region as the mirrored mask from the border (Fig. 4a). The second comprises a bounding box centered at the ground roll mask and another one with the same size displaced horizontally towards the signal area (to compare similar reflectors), as depicted in Fig. 4b.

Following Oliveira et al.^28^, we used the power spectrum to evaluate signal and noise frequencies, histograms for evaluating their amplitude distributions, and the noise detection model outcomes for assessing noise residuals. Each of these aspects derived specific metrics for evaluating over and under attenuation in the filtered area, as we describe below.

High amplitude values characterize ground roll noise, and we propose to evaluate the amplitude values in one of our metrics. We compute the amplitudes histogram for noise-free and filtered areas and derive a metric (*F*1) considering the locus depicted in Fig. 4a. We compute two histograms: *H*(*S*) from a signal region and *H*(*N*) from a ground roll-affected area. Our metric is then given by:

2 $$\begin{aligned} F1 = \sum \limits _{i=min}^{p_1} (H(N)_i - H(S)_i) + \sum \limits _{i=p_2}^{max} (H(N)_i - H(S)_i) \end{aligned}$$

where $p_1,p_2$ are the 10th and 90th percentile are considered as the ground roll high-amplitude characteristic values. We set *min* and *max* as the minimum and maximum amplitudes observed in the normalized shot gather evaluated.

*F*1 metric ranges between $[-1,1]$, where values close to $-1$ are likely to be *over* attenuated, and values close to 1 are likely to be *under* attenuated. Successful ground roll suppression results in very similar histograms, which is considered the target for both over and under attenuation scores. Over attenuated gathers would present few pixels with very high amplitudes and therefore negative *F*1 values, while under attenuated gathers would present very high amplitudes in the ground roll affected regions and, therefore, high positive *F*1 values.

The second metric (*F*2) refers to activation values from the noise detection network presented by Oliveira et al.^28^ The evaluation locus used is defined in Fig. 4a. We compute the difference between the average detection value for the ground roll affected area and the one in the signal region with low activation values. High *F*2 values suggest there is still identifiable ground roll noise in the filtered region, and low *F*2 values indicate noise activation values comparable to those observed in the signal. Negative *F*2 values reflect highly over attenuated areas, where the activation values are even lower than those seen in signal areas.

Our last metric concerns frequency analysis, and for that, we compare the power spectrum periodograms for noise-free and filtered areas. *F*3 considers the evaluation locus defined in Fig. 4b and disregards differences between over and under attenuation. We compute two power spectrum periodograms—*P*(*N*) and *P*(*S*)—the first one considering a set of noise bounding boxes, and the second using signal bounding boxes. The difference between those two periodograms should be minimal in case of successful filtering. *F*3 is formally defined as the proportion of energy considering the difference between *P*(*N*) and *P*(*S*) in frequency range between 5Hz and 60Hz:

3 $$\begin{aligned} F3 = 1-\frac{\sum \nolimits _{i=5}^{60} abs(P(S)_i - P(N)_i)}{\sum \nolimits _{i=5}^{60} abs(P(S)_i + P(N)_i)} \end{aligned}$$

Since we evaluate our results considering over and under attenuation, we combine the metrics differently with respect to each target. The final over ($F_o$) and under ($F_u$) attenuation scores are computed as the average of our three metrics after different normalization setups for each target, as explained below. To compute $F_o$, we map *F*1 and *F*2 linearly from the interval $[-1,0]$ to the score [0, 100], multiply *F*3 by 100, and average the three metrics. This means that over attenuated filtered regions would have highly negative *F*1 and *F*2 values and a low *F*3 value, resulting in low $F_o$ score values. For $F_u$, we map *F*1 and *F*2 scores linearly from the interval [0, 1] to the score [100, 0], multiply *F*3 by 100 and average them. Under attenuated filtered regions would have highly positive *F*1 and *F*2 values and a low *F*3 value, resulting in low $F_u$ score values.

**Evaluate QC**: Similar to the VA use case, we adopted the K-means strategy to define the groups *good*, *average*, and *bad*. In the NS geocycle, however, the score to be evaluated depends on the cycle currently being executed. If the purpose of the cycle is to improve over attenuation, then we use $F_o$ for clustering. Otherwise, we use $F_u$.

**Select samples for the next cycle**: We followed the same idea as in the VA use case to select which samples should be re-evaluated in the next cycle, i.e., only those in the groups *average* and *bad*.

## Results and discussion

In the following sections, we present and discuss the results for each experiment, focusing on the evolution of QC scores and the number of gathers in each group. We have performed experiments for two distinct datasets named DS1 and DS2. For the sake of brevity, and since the results led to similar conclusions, we decided to present a detailed analysis for DS2 only.

### Velocity analysis

As mentioned in the previous section, in step 7 of the VA pipeline (Fig. 2—Evaluate QC scores), we used a clustering algorithm (K-means) to separate the QC scores into three groups: *good*, *average*, and *bad* results. In the first cycle, we fit the K-means algorithm to the QC score values, defining our scale. In the next cycles, the fitted K-means is used to decide in which group the current QC scores should be placed. The average scores of the clusters were 48.82, 68.62 and 92.45 for *good*, *average*, and *bad*, respectively. Table 1 shows how many gathers were in each group for each cycle. It took six cycles to meet the stopping condition.

**Table 1:** Number of gathers classified as good, average, or bad in each cycle of the experiment for DS2. The numbers are also given as percentages from the total number of gathers.

Cycle	Good		Average		Bad		Sum	
	# gathers	% from total	# gathers	% from total	# gathers	% from total	# gathers	% from total
1	31,232	37.03	35,836	42.48	17,282	20.49	84,350	100.00
2	23,975	28.42	19,592	23.23	9551	11.32	53,118	62.97
3	7855	9.31	13,858	16.43	7430	8.81	29,143	34.55
4	4970	5.89	10,814	12.82	5504	6.53	21,288	25.24
5	2581	3.06	7543	8.94	6194	7.34	16,318	19.35
6	853	1.01	4572	5.42	8312	9.85	13,737	16.29
Total gathers	84,350

**Table 2:** Number of gathers in each group considering the complete QC score (updated in every cycle) for experiment DS2. The numbers are also given as percentages from the total number of gathers.

Cycle	Good		Average		Bad	
	# gathers	% from total	# gathers	% from total	# gathers	% from total
1	31,232	37.03	35,836	42.48	17,282	20.49
2	55,207	65.45	24,391	28.92	4752	5.63
3	63,062	74.76	18,735	22.21	2553	3.03
4	68,032	80.65	14,787	17.53	1531	1.82
5	70,613	83.71	12,641	14.99	1096	1.30
6	71,466	84.73	11,856	14.06	1028	1.22
Total gathers	84,350					

In the first cycle, 37% of the total number of gathers we considered in DS2 were classified as good and 20.5% as bad. This result indicates that the first gathers selected for training were somewhat representative of the data. In other words, the patterns that the network learned from these gathers were enough to produce good scores for 37% of the data in DS2.

Once a gather is placed in the cluster good, it is not considered in the subsequent cycles anymore. Only the ones in the other groups continue in the process. One can observe this by examining the last column of Table 2: the sum of processed gathers represents a decreasing percentage of the total number of gathers. In this experiment, the number of gathers moving to the cluster good was our stopping condition: in cycle 6, less than 3% of total gathers were classified as good, so the process stopped.

**Figure 5:**
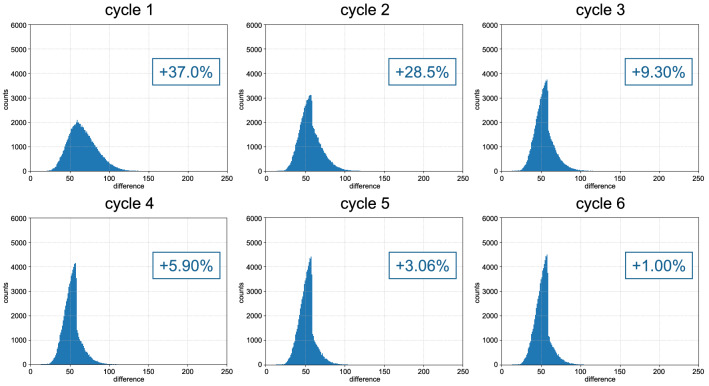
Evolution of the difference score histogram for DS2. The blue boxes show the percentage of the total gathers that went to the group good in each cycle.

Comparing the columns average and bad in Table 1, one can note that, in some cycles, we have more average gathers than bad, and in others, the opposite. For example, imagine that gather *X* is classified as average in cycle 2. In cycle 3, we will train another network with other gathers and adjust the velocity of gather *X* again. Its score in cycle 3 can be worse than before, as the new model is not necessarily better for gather *X*. Therefore, the gathers that stay in the clusters *average* and *bad* can move between them in different cycles. However, we keep track of the best scores and corresponding velocities, that is, the overall scores never get worse. If gather *X* gets a difference score of 40 in cycle 2 and 60 in cycle 3, its score and velocity are not updated in cycle 3. Figure 5 shows the evolution of the overall difference score for DS2.

Notice in Fig. 5 that the left part of the histogram increases with the progression of cycles, showing that the difference values are decreasing. At the end of the process, the histogram is considerably thinner than at the beginning.

To further investigate the evolution of the QC score values, we used the fitted K-means to classify the overall QC scores (updated only with improved values) into the three groups. In this way, we can see not only how many gathers go to the group good, but how many actually stay in the other two. Table 2 shows the number of gathers in each group for the overall score and the percentage of the total gathers the numbers represent. Note that all rows in Table 3 sum 100%, as we are evaluating the overall score, which includes all gathers. From Table 2, it is possible to see that more than 84% of the gathers ended up in the group *good*, while only 1.22% stayed in the *bad*. This information complements the histograms of Fig. 5, showing that the overall score improved considerably with the progression of the cycles.

**Figure 6:**
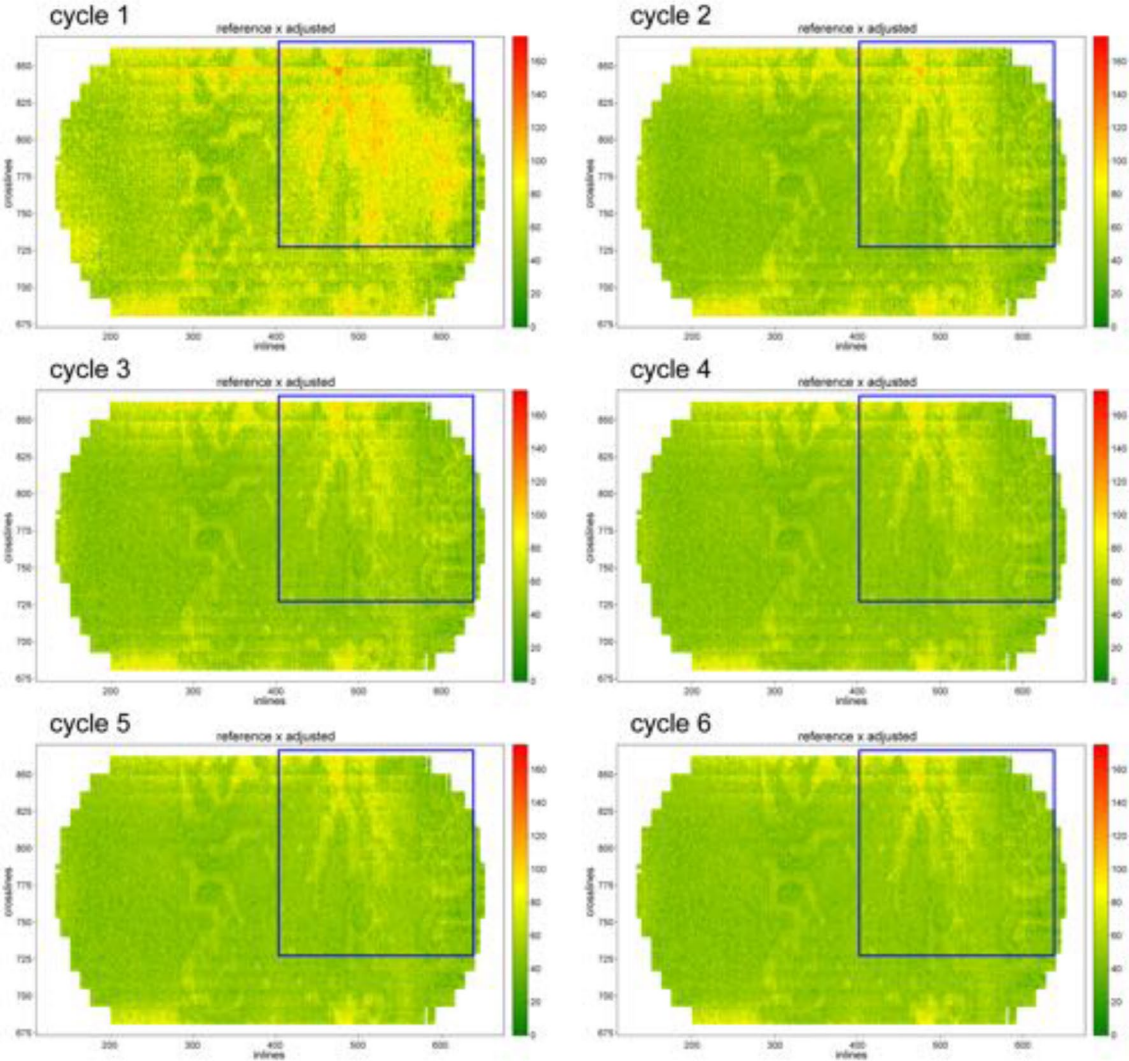
Evolution of the difference score for the gathers of DS2 with a colormap: the greener, the better. The horizontal axis represents the inlines, and the vertical, the crosslines. The blue box highlights a region where the results were considerably improved.

In Fig. 6, we present the overall difference QC score for each gather with a colormap, to investigate how the scores are spread throughout the cube. In the colormap, dark green means better score values, while yellow and red correspond to worse values. In the first cycle, there is a large yellow area on the right, and green spots are quite dispersed, indicating that the first list of training gathers may not have been representative for some parts of the dataset. However, we can see in Fig. 6 that the number of green spots increases throughout the cycles, showing that the results are improving. Some regions remain yellowish until the end. This might indicate that there are particularities in some regions of the cube, and they may have to undergo a visual quality check or even need a specific processing step.

**Table 3:** Geocycles tabular results for noise suppression. We list the proportion as percentages of gathers classified as good, average, or bad for over and under attenuation criteria in each cycle. We also list the average scores for both criteria and the percentage of gathers evaluated in each cycle.

Cycle	% Good		% Average		% Bad		% Avg. score		% Evaluated
	$F_o$	$F_u$	$F_o$	$F_u$	$F_o$	$F_u$	$F_o$	$F_u$	–
1-Initial	50.13	61.90	32.75	30.84	17.13	7.26	71.5	96.28	100.00
2-Over	81.86	10.88	14.02	18.73	4.13	70.39	74.7	83.48	49.88
3-Over	82.08	10.81	13.86	18.78	4.05	70.41	74.71	83.4	18.15
4-Over	82.95	10.70	12.87	17.51	4.18	71.79	74.75	83.0	17.91
5-Under	83.74	16.69	12.16	52.34	4.10	30.96	74.92	91.84	100.00
6-Under	83.33	18.78	13.28	48.57	3.39	32.65	74.89	91.48	83.3
Total gathers	3924

### Ground roll noise suppression

**Figure 7:**
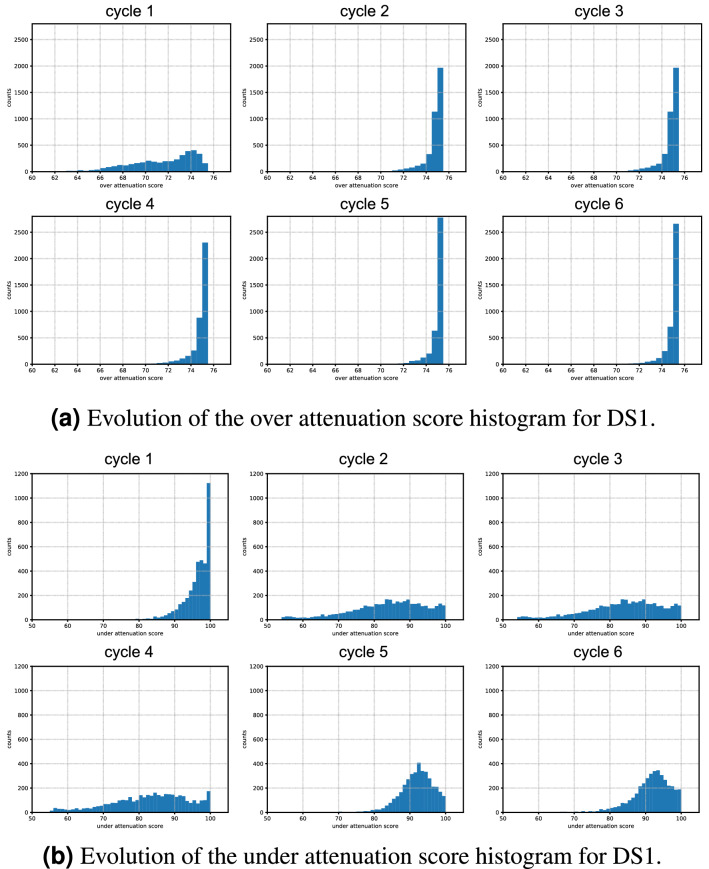
Histograms of over and under attenuation scores for each cycle.

We executed three cycles using $F_o$, for improving over attenuation, and two cycles using $F_u$ in the sequence for improving under attenuation, considering that the first cycle evaluated as over attenuated, as cycle-1 in Fig. 7a, b indicate.

For the first cycle, we selected a regular grid with 10 gathers to compose our first training set. We trained the first model, as depicted in step 2 in Fig. 3, and evaluated the full dataset. We compute noise suppression QC scores in step 4 and evaluate them in step 5, as depicted in Fig. 3, and cluster them into three groups: good, average, and bad. In the first cycle, we evaluate the QC score values and define our scale, used in the next cycles to decide the quality group the current samples should be placed.

It is important to notice that our scales for over and under attenuation will consider different criteria for indicating a sample result as good, average or bad. Since the first execution evaluation points to a better evaluation for under attenuation (96.28 average score) than to over attenuation (71.5 average score), the scale for over attenuation is softer compared to under attenuation. This effect could be alleviated by dynamically changing the scale, or defining it heuristically, but in our experiments we considered the initial scales, so we could compare the evolution through the cycles.

For the first cycle, the average scores for over and under attenuation are 96.28 and 71.5, respectively. The proportions for *good*, *average*, and *bad* clusters were 50.13%, 32.75% and 17.13% for over attenuation and 61.90%, 30.85% and 7.26% for under attenuation. Table 3 details the same metrics for each cycle, considering over and under attenuation, and it is possible to identify an evolution towards better over attenuation scores, for over attenuation cycles, and better under attenuation scores, for under attenuation cycles, as expected. We also observe that a consequent migration of samples to different quality clusters happens accordingly.

We further present in Table 3 the amount of gathers considered for testing and evaluation, which is the sum between the average and bad quality samples from the previous cycle. For over attenuation, we observe that these proportions decreased from initial 100% to around 18%, which is a reduction of nearly 5 times. For under attenuation cycles, since our scale is tougher, this effect is not as impressive, and we reduced from the initial 100% in the first under attenuation cycle, to 83.3% in the second. It is important to notice that reducing the amount of gathers to be tested is directly related to saving computational processing time and resources.

Figure 7 visually presents the same evolution detailed in Table 3, and we can observe a clear evolution for the over attenuation scores in the first cycles, and a clear evolution of under attenuation scores in the last cycles. We also observed a “memory” effect during training, i.e., the network was able to improve results in the last two cycles for under attenuation, while still delivering good metrics for over attenuation, even if the criteria for selecting training samples disregarded over attenuation scores for those two last cycles.

**Figure 8:**
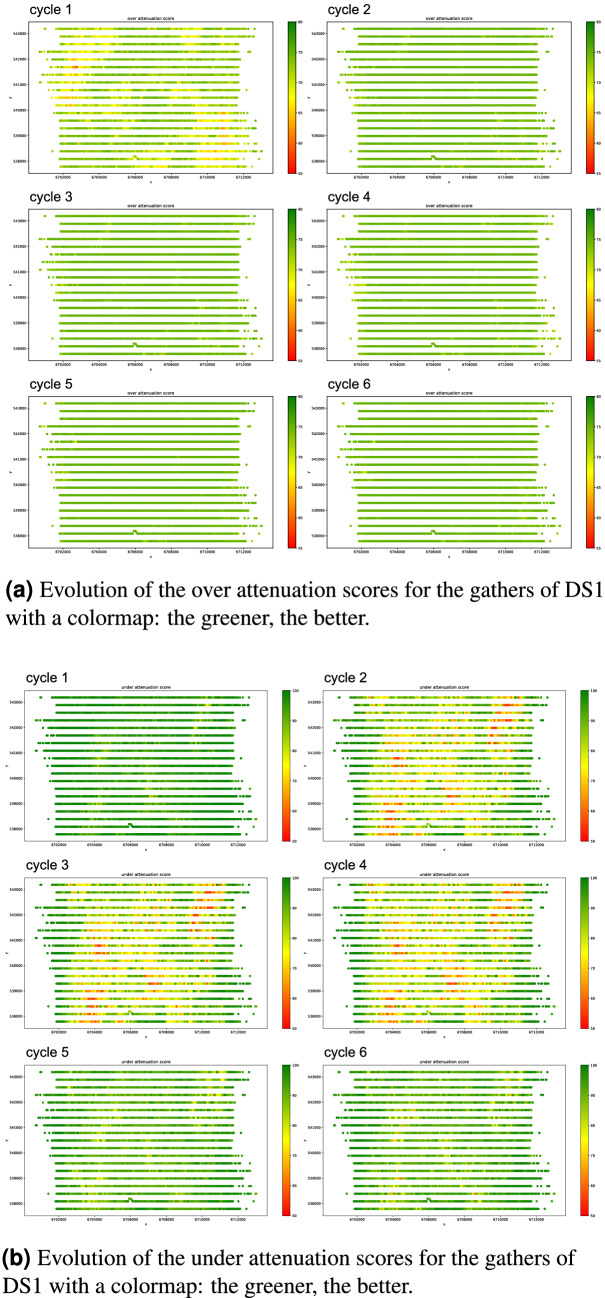
Spatial distribution of over and under attenuation scores through the cycles for DS1.

Additionally, we present the spatial distribution of scores for both over and under attenuation in Fig. 8. It is interesting to observe that poorly evaluated gathers form clusters around specific regions, which indicates that those gathers had specific acquisition or geological conditions that were not contemplated in the first training set, and therefore presented poor results in testing. During the cycles, we observe a quality increase in those clusters, as we hypothesized, which supports that the proposed cyclic model is able to achieve overall good quality for seismic processing in large areas.

## Conclusion

In this paper, we presented a cyclic learning approach called Geocycles to improve seismic processing tasks using machine learning. The methodology comprises some consistent steps: (1) creating a training set, (2) choosing an adequate machine learning method for a given task, (3) training the machine learning model, (4) processing the data with the trained model, (5) computing metrics consistent with desired characteristics in the outcome, (6) assessing the metrics quantitatively and (7) selecting training samples for the next cycle.

We further built on two typical use cases in seismic processing and created specific geocycles for each of them. First, we presented an example of velocity analysis task that showed consistently improved results through cycles. Second, we introduced a geocycle for suppressing ground roll noise in pre-stack images and two different target setups for over and under attenuation. Our results were again consistently improved through the cycles, and we believe that the results of both use cases support the hypothesis that we raised in this paper. Our method enables an iterative optimization scheme that could deliver optimal overall results for large areas compared to only training a single model, as observed in the first cycle.

As future research, we consider the composition of cycles to cover sequential seismic processing tasks. Also, we intend to explore geocycles comprising different tasks that could be evaluated to improve each other. For instance, considering the selected use cases, we could think of connections after velocity picking as feedback for improving noise attenuation at specific underperforming areas. Additionally, we envisage that the proposed methodology could work with a human in the loop at each cycle or triggered at specific convergence points, even though we explored only automatic workflow use cases. Other research directions point to evaluating the methodology in other seismic processing tasks and using datasets from different geological contexts.
